# Patient-reported outcome (PRO) results from the AGITG DOCTOR trial: a randomised phase 2 trial of tailored neoadjuvant therapy for resectable oesophageal adenocarcinoma

**DOI:** 10.1186/s12885-022-09270-4

**Published:** 2022-03-15

**Authors:** R. Mercieca-Bebber, E. H. Barnes, K. Wilson, Z. Samoon, E. Walpole, T. Mai, S. Ackland, M. Burge, G. Dickie, D. Watson, J. Leung, T. Wang, R. Bohmer, D. Cameron, J. Simes, V. Gebski, M. Smithers, J. Thomas, J. Zalcberg, A. P. Barbour

**Affiliations:** 1 National Health and Medical Research Council (NHMRC) Clinical Trials Centre, Faculty of Medicine and Health, University of Sydney, Sydney, NSW Australia; 2 Division of Cancer Services, Princess Alexandra Hospital, Woolloongabba, Qld Australia; 3 School of Clinical Medicine, University of Queensland, Brisbane, Qld Australia; 4 Faculty of Medicine, The University of Queensland, Brisbane, Qld Australia; 5 School of Medicine and Public Health, University of Newcastle, Newcastle, NSW Australia; 6 Cancer Care Services, Royal Brisbane and Women’s Hospital, Brisbane, Qld Australia; 7 Discipline of Surgery, College of Medicine and Public Health, Flinders University, Adelaide, South Australia; 8 GenesisCare St Andrew’s Hospital, 352 South Terrace, Adelaide, SA Australia; 9 Crown Princess Mary Cancer Center, Westmead hospital; Faculty of Medicine and Health, University of Sydney, Sydney, Australia; 10 Hobart Private Hospital, Ground Floor- Suite 6 Corner Argyle & Collins Streets, Hobart, Tasmania Australia; 11 Townsville University Hospital, Townsville, Qld Australia; 12 Divisions of Surgery and Cancer Services, Princess Alexandra Hospital, Woolloongabba, Australia; 13 GIAST Clinic Mater Medical Centre South Brisbane, Brisbane, Australia; 14 School of Public Health and Preventive Medicine, Monash University, Melbourne, Australia

**Keywords:** Oesophageal adenocarcinoma, Gastro-oesophageal adenocarcinoma, Neoadjuvant therapy, Quality of life, Patient-reported outcomes

## Abstract

**Background:**

AGITG DOCTOR was a randomised phase 2 trial of pre-operative cisplatin, 5 fluorouracil (CF) followed by docetaxel (D) with or without radiotherapy (RT) based on poor early response to CF, detected via PET, for resectable oesophageal adenocarcinoma. This study describes PROs over 2 years.

**Methods:**

Participants (*N* = 116) completed the EORTC QLQ-C30 and oesophageal module (QLQ-OES18) before chemotherapy (baseline), before surgery, six and 12 weeks post-surgery and three-monthly until 2 years. We plotted PROs over time and calculated the percentage of participants per treatment group whose post-surgery score was within 10 points (threshold for clinically relevant change) of their baseline score, for each PRO scale. We examined the relationship between Grade 3+ adverse events (AEs) and PROs. This analysis included four groups: CF responders, non-responders randomised to DCF, non-responders randomised to DCF + RT, and “others” who were not randomised.

**Results:**

Global QOL was clinically similar between groups from 6 weeks post-surgery. All groups had poorer functional and higher symptom scores during active treatment and shortly after surgery, particularly the DCF and DCF + RT groups. DCF + RT reported a clinically significant difference (−13points) in mean overall health/QOL between baseline and pre-surgery. Similar proportions of patients across groups scored +/− 10 points of baseline scores within 2 years for most PRO domains. Instance of grade 3+ AEs were not related to PROs at baseline or 2 years.

**Conclusions:**

By 2 years, similar proportions of patients scored within 10 points of baseline for most PRO domains, with the exception of pain and insomnia for the DCF + RT group. Non-responders randomised to DCF or DCF + RT experienced additional short-term burden compared to CF responders, reflecting the longer duration of neoadjuvant treatment and additional toxicity. This should be weighed against clinical benefits reported in AGITG DOCTOR. This data will inform communication of the trajectory of treatment options for early CF non-responders.

**Trial registration:**

Australia New Zealand Clinical Trials Registry (ANZCTR), ACTRN12609000665235. Registered 31 July 2009.

**Supplementary Information:**

The online version contains supplementary material available at 10.1186/s12885-022-09270-4.

## Key message

Participants with oesophageal adenocarcinomas who did not respond to early CF and were randomised to pre-operative DCF + RT experienced a short-term clinically-relevant detriment to PROs, which by 2 years post-surgery, largely returned to baseline levels. This was comparable to those randomised to DCF alone, and early CF responders.

## Background

Adenocarcinomas of the oesophagus and gastro-oesophageal junction affect the glandular cells of the mucosa, from the lower part of the oesophagus to where the oesophagus joins the stomach. These oesophageal glands produce mucus to assist the passage of swallowed food in digestion. The disease and its treatment can cause these cells to malfunction, resulting in dysphagia, dry mouth, pain, altered taste, trouble eating, reflux or speech problems, which can impact overall quality of life [1].

Treatment for those with potentially curable disease typically involves surgery with pre-operative (neoadjuvant) chemotherapy or chemo-radiotherapy (CRT). Those who respond to chemotherapy tend to have more favourable prognosis [2–4]. Meta-analyses have found that neoadjuvant CRT, or chemotherapy, as compared to surgery alone for oesophageal adenocarcinoma offer an overall survival benefit [5–9]. However, direct comparisons of these two approaches do not show a consistent benefit for one over the other [10] and complication rates may be higher following nCRT [9]. There are few published reports with patient-reported outcome (PRO) data from randomized neoadjuvant therapy trials, representing a gap in our understanding of the impact of neoadjuvant therapy [11]. Recent trials have focused on identifying suitable candidates for neoadjuvant therapy regimens, involving chemotherapy or CRT. The Australasian Gastro-Intestinal Trials Groups (AGITG) DOCTOR trial [12] was a randomised phase 2 trial of pre-operative cisplatin, 5 fluorouracil (CF) and docetaxel (D) with or without radiotherapy (RT) based on poor early response to standard chemotherapy for resectable adenocarcinoma of the oesophagus and/or oesophageal junction [12].

### The AGITG DOCTOR trial

**Fig. 1:**
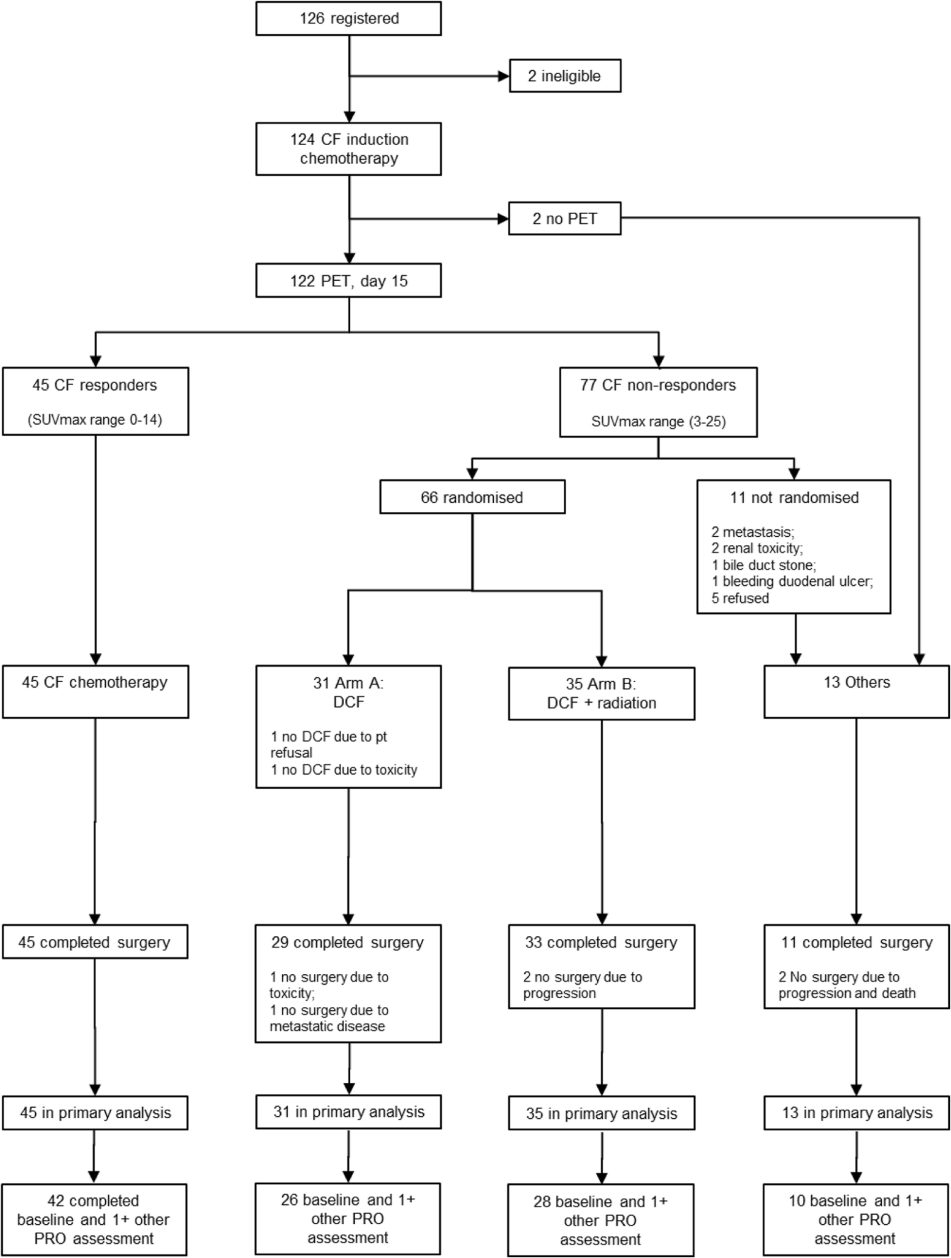
Flow of participants in the AGITG DOCTOR Trial

Participants (*N* = 124) were registered to the AGITG DOCTOR trial between 8 July 2009 and 29 December 2015 [12]. Figure 1 depicts the flow of participants into four treatment groups: early responders, non-responders randomised to DCF, non-responders randomised to DCF + RT, and “others” excluded from randomisation or PET scan [12]. Major histological response, the primary trial endpoint, was achieved for 3/45 (7%) of the early responder group, 6/30 (20%) of the DCF group, 22/35 (65%) of the DCF + RT group, and 0/13 (0%) of “others” [12]. Three-year progression-free survival rates were 47% in the early responder group, 29% in the DCF group, 46% in the DCF + RT group and 37% for “others”. Five-year overall survival was 53% for early responders, 31% for DCF arm, 46% for DCF + RT, and 35% for “others” [12]. The results indicated that early PET/CT has potential utility for tailoring therapy for early non-responders to CF, and these patients benefit from the addition of docetaxel and RT [12].

The additional toxicity associated with docetaxel and RT was evident by the higher incidence of grade 3–4 adverse events in the DCF and, particularly in the DCF + RT, groups. In fact, 42 and 71% of participants respectively experienced at least one grade 3–4 toxicity, compared to 27% of the early responder group and 46% of the “others” [12]. All participants in the DCF and DCF + RT groups experienced an adverse event at any grade [12]. Undoubtedly, these effects would have impacted quality of life and functional outcomes. The extent of this impact requires investigation in order to inform future patients about the possible burden associated with treatment options.

This study aims to: 1) describe key PROs of the AGITG DOCTOR trial over time; 2) state proportions of participants who returned to baseline levels of PRO scores; 3) determine if the instance of a Grade 3–4 adverse event impacted long term health/QOL, physical functioning or role functioning at 2 years; and 4) determine if a relationship exists between poorer overall QOL/health score at baseline and instance of a Grade 3–4 AE at any point in the 2 year period.

## Methods

Details of patient selection and the treatment protocol have been reported previously [12]. Participants completed the European Organisation for Research and Treatment of Cancer (EORTC) Quality of Life Questionnaire Core 30 (QLQ-C30) and oesophageal cancer module (QLQ-OES18) questionnaires in hard-copy at baseline (up to 28 days before commencing initial chemotherapy), before surgery (up to 14 days prior), 6 and 12 weeks post-surgery (+/− 2 weeks), 12 weeks post-surgery, and three-monthly until 2 years (+/− 4 weeks). These questionnaires were selected by our multidisciplinary research team because they were developed using high quality methods, have evidence supporting their validity [1, 13, 14] and cover important issues relevant to the study population, particularly issues associated with swallowing function. The 2 year time point is clinically important because previous research has demonstrated that 82% of disease recurrences occur within 2 years [15].

The QLQ-C30 has 30 items and a total of 15 sub-scales: five functioning scales (physical, role, emotional, cognitive, social), three symptom scales (fatigue, nausea/vomiting, pain), six single item scales (dyspnoea, insomnia, appetite loss, constipation, diarrhoea, financial difficulties) and a global health status/quality of life (QOL) scale [13, 16]. The QLQ-OES18 four scales (dysphagia, eating, reflux, oesophageal pain) and six single items (swallowing saliva, choking when swallowing, dry mouth, taste problems, coughing and speech problems) [1]. The questionnaires ask participants to report on their health in the past week using a four point Likert scale, and seven-point scale for overall health and quality of life.

### Analysis

The EORTC QLQ-C30 was scored according to the manual [16] using all available data. Missing items were imputed using the scale mean, if more than 50% of the scale had been completed, as per the scoring manual. Mean and range scale scores and were calculated for each analysis group and plotted for each time point, by analysis group. Changes in PRO scores of more than 10 points were interpreted as clinically significant [17]. No statistical between-group comparisons were made. The mean and 95% confidence intervals (CI) for the two randomised groups were also plotted over time for key PRO scales (physical functioning, role functioning, fatigue, pain, global health/ QOL, dysphagia, trouble swallowing, choked when swallowing), selected a priori. We also calculated the percentage of participants per treatment group whose post-surgery score was no more than 10 points (threshold for clinically relevant change) worse than their baseline score, for each PRO scale. Swallowing scales of the QLQ-OES18; specifically dysphagia, eating, oesophageal pain, swallowing saliva, choking when swallowing, and dry mouth; were identified a priori as key scales for this analysis. Participants who completed baseline and at least one subsequent PRO assessment were included.

All participants were combined to assess whether patient-reported overall health/QOL, physical or role functioning and other PRO domains at baseline predicted the instance of a grade 3 or 4 adverse event (yes/no) using logistic regression analysis. PRO domains at 2 years were compared between participants who had and had not experienced a grade 3 or 4 adverse event using the Wilcoxon rank sum test.

Questionnaire completion rates were calculated for each of the four groups, at each time point. The completion rate was defined as the number of completed questionnaires available for analysis, divided by the number of questionnaires expected at that time point, multiplied by 100. Thus, questionnaires were not expected from participants who had died or were withdrawn from the study.

The reporting of this study adheres to CONSORT-PRO guidelines [18].

## Results

### PRO completion rates

One-hundred-and-sixteen of 124 (93.5%) participants included in the main study participated in this PRO study. PRO questionnaire completion rates fluctuated at each time point, ranging from 76 to 100% overall at each time point (Additional file 1). A 100% baseline questionnaire completion rate was achieved. Completion rates were poorest at the 6 week follow up, particularly for DCF and DCF + RT groups, with 67 and 65% completion respectively. The “others” group had the poorest completion rates overall. In a post-hoc comparison, there was no evidence that non-completion at 6 weeks was associated to any baseline differences in PROs (Additional file 1).

Patient characteristics, including baseline PRO scores, are reported in Table 1.

**Table 1:** patient characteristics, including survival outcomes

Characteristic	PET responders: CF (***N*** = 45)	PET non-responders randomised: DCF (***N*** = 31)	PET non-responders randomised: DCF + radiation (***N*** = 35)	Others (***N*** = 13)	All patients (***N*** = 124)
Age in years, median (range)	61 (42–78)	60 (38–77)	63 (44–76)	65 (41–71)	63 (38–78)
Male	41 (91)	27 (87)	32 (91)	10 (77)	110 (89)
ECOG 0	37 (82)	25 (81)	27 (77)	13 (100)	102 (82)
Days between start of chemotherapy and surgery, mean (SD)	69 (10)	117 (19)	126 (24)	57 (22)	
PFS in months, Median (95% CI)	27 (12–.)	22 (14–27)	22 (14–.)	13 (5–.)	22 (15–36)
OS in months, Median (95% CI)	61 (26–.)	30 (17–55)	35 (20–.)	31 (5–.)	36 (25–61)
**Baseline QLQ-C30 and QLQ-OES18 scores per scale, mean (SD)**					
Physical functioning	94 (10)	94 (12)	96 (9)	89 (13)	94 (10)
Role functioning	88 (22)	82 (29)	90 (16)	85 (20)	87 (22)
Emotional functioning	76 (23)	74 (21)	74 (19)	81 (16)	75 (21)
Cognitive functioning	86 (17)	91 (20)	89 (14)	94 (11)	89 (16)
Social functioning	78 (28)	75 (29)	80 (23)	79 (26)	78 (27)
Fatigue	19 (20)	24 (20)	19 (19)	25 (21)	21 (20)
Nausea and vomiting	8 (14)	16 (26)	11 (14)	12 (17)	11 (18)
Pain	17 (21)	15 (22)	14 (16)	26 (19)	17 (20)
Dyspnoea	10 (15)	9 (22)	7 (18)	8 (15)	9 (18)
Insomnia	29 (33)	22 (29)	24 (26)	36 (29)	27 (30)
Appetite loss	17 (23)	23 (30)	24 (23)	31 (29)	22 (26)
Constipation	12 (22)	14 (19)	13 (18)	23 (34)	14 (22)
Diarrhoea	5 (14)	5 (12)	3 (10)	5 (13)	5 (12)
Financial difficulties	23 (24)	26 (31)	29 (31)	18 (29)	25 (28)
Global health status/QoL	74 (18)	75 (15)	72 (16)	74 (15)	74 (16)
Dysphagia	22 (23)	27 (20)	25 (23)	21 (19)	24 (22)
Problems with eating	27 (24)	40 (28)	38 (27)	46 (31)	35 (27)
Reflux	17 (18)	23 (28)	21 (27)	27 (24)	21 (24)
Pain (OES-18)	22 (23)	21 (22)	23 (21)	23 (18)	22 (21)
Trouble swallowing saliva	13 (24)	17 (28)	14 (28)	15 (26)	14 (26)
Choked when swallowing	9 (15)	12 (28)	21 (28)	13 (22)	14 (23)
Dry mouth	12 (18)	35 (33)	19 (30)	13 (22)	19 (27)
Trouble with taste	4 (13)	9 (20)	16 (28)	10 (16)	9 (20)
Trouble with coughing	9 (21)	15 (27)	11 (22)	8 (15)	11 (22)
Trouble with talking	3 (10)	4 (14)	6 (20)	5 (13)	4 (14)

### Key PROs over time

**Fig. 2:**
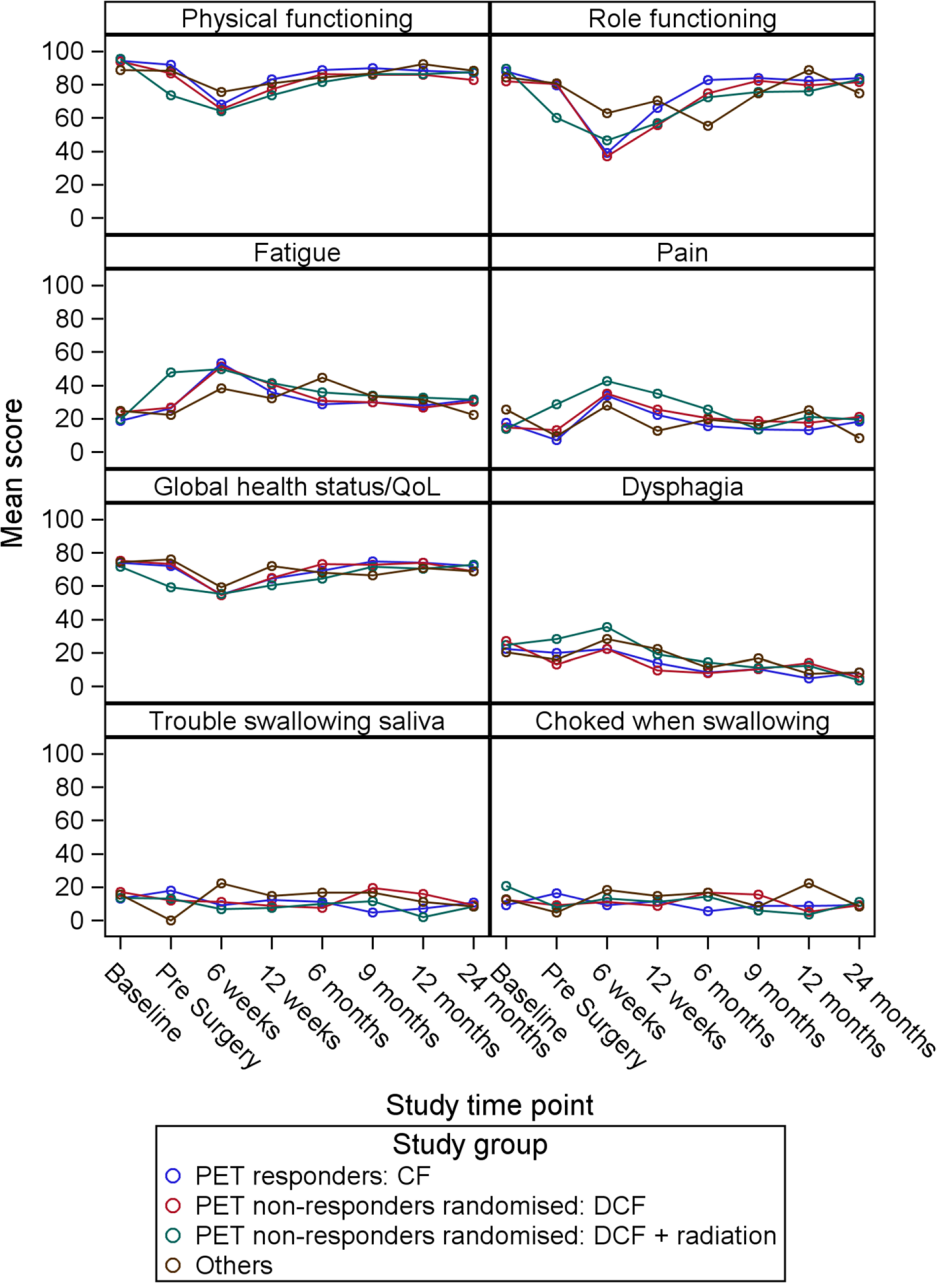
Mean scale scores for key QLQ-C30 functional scales and key QLQ-OES18 swallowing scales until 24 months post-surgery

Figure 2 shows mean scale scores for key functional scales of the QLQ-C30 and key swallowing scales of the QLQ-OES18 until 24 months post-surgery (remaining scales are presented in Additional file 2). All groups had poorer functional and higher symptom scores before, and 6-weeks after, surgery - particularly the DCF and DCF + RT groups. During this period, DCF + RT reported a clinically significant mean difference (−13points) in overall health/QOL, a decline in mean score of more than 10 points across all QLQ-C30 functional scales, as well as increases of similar magnitudes in symptoms of pain (QLQ-C30), fatigue, and dyspnoea. There were smaller (< 10 point) fluctuations in mean scales scores for other groups at these time points.

All treatment groups experienced a decline in mean score of more than 10 points in physical and role functioning, and overall health/QOL between baseline and 6-weeks-post-surgery, along with increases of more than 10 points in mean pain (QLQ-C30), nausea/vomiting, dyspnoea, appetite loss and coughing. Broadly, these issues resolved over the 2 years of follow up. Global QOL was clinically similar between groups from 6 weeks post-surgery.

**Fig. 3:**
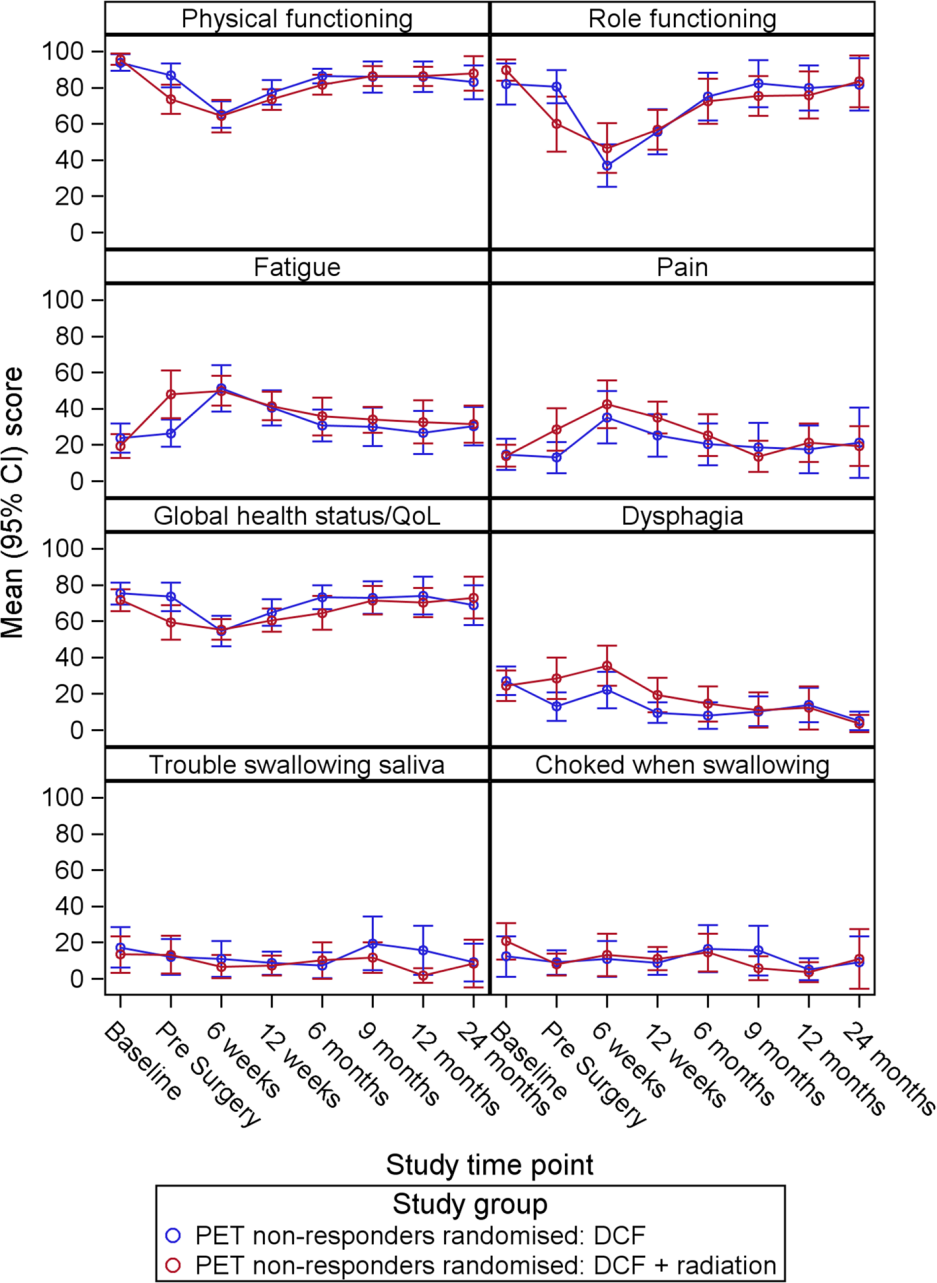
Mean and 95% CI over time for participants randomised to DCF alone VS DCF+ RT

Figure 3 shows mean PROs over time for early non-responders who were randomised to DCF versus DCF + RT only (i.e. early responders and the “other” group are excluded) for key PRO scales. The participants randomised to DCF + RT experienced lower physical and role functioning, worse global QOL, higher levels of fatigue, pain, and dysphagia at the pre-surgery time point, however the only scale where confidence intervals did not overlap at this time point was fatigue. Mean scores for most of these scales were similar by 6 weeks post-surgery, however pain and dysphasia appeared worse, on average, for participants randomised to DCF + RT until 12 weeks.

### PRO recovery as compared to baseline levels

Table 2 shows that most patients returned to within +/− 10 points of their baseline scores for each scale at some point during the 24 months of post-surgical follow up. Eighteen participants were excluded from this analysis as they had insufficient data. The scales where fewest participants overall returned to baseline levels were role functioning and fatigue, for which 66 and 55% of participants returned to within 10 points of their baseline levels, respectively. The rate of participants who recovered to within +/− 10 points of baseline scores was notably lower for the DCF + RT group for domains of insomnia and pain, compared to other groups, however no statistical comparisons were made due to the non-comparative study design.

**Table 2:** PRO recovery to near baseline

		n (%) who recover to within 10 points of baseline after surgery (where QoL data available)				
QoL imeasure	QoL subscale	PET responders: CF	PET non-responders randomised: DCF	PET non-responders randomised: DCF & RTT	Others	All patients
	N	**42**	**26**	**28**	**10**	106
QLQ-C30	Physical functioning	33 (79)	18 (69)	18 (64)	10 (100)	79 (75)
	Role functioning	28 (67)	19 (73)	17 (61)	6 (60)	70 (66)
	Emotional functioning	37 (88)	25 (96)	25 (89)	10 (100)	97 (92)
	Cognitive functioning	35 (83)	20 (77)	20 (71)	7 (70)	82 (77)
	Social functioning	34 (81)	18 (69)	22 (79)	8 (80)	82 (77)
	Fatigue	23 (55)	16 (62)	14 (50)	5 (50)	58 (55)
	Nausea and vomiting	33 (79)	19 (73)	20 (71)	7 (70)	79 (75)
	Pain	36 (86)	23 (88)	19 (68)	9 (90)	87 (82)
	Dyspnoea	32 (76)	17 (65)	18 (64)	8 (80)	75 (71)
	Insomnia	39 (93)	22 (85)	19 (68)	10 (100)	90 (85)
	Appetite loss	36 (86)	22 (85)	25 (89)	7 (70)	90 (85)
	Constipation	39 (93)	26 (100)	26 (93)	8 (80)	99 (93)
	Diarrhoea	39 (93)	20 (77)	26 (93)	10 (100)	95 (90)
	Financial difficulties	37 (88)	24 (92)	25 (89)	10 (100)	96 (91)
	Global health status	28 (67)	18 (69)	20 (71)	8 (80)	74 (70)
	Global Quality of life	30 (71)	18 (69)	18 (64)	7 (70)	73 (69)
	Global health status/QoL	30 (71)	20 (77)	21 (75)	8 (80)	79 (75)
OES-18	Dysphagia	40 (95)	23 (88)	25 (89)	8 (80)	96 (91)
	Problems with eating	33 (79)	26 (100)	23 (82)	9 (90)	91 (86)
	Reflux	32 (76)	22 (85)	22 (79)	8 (80)	84 (79)
	Pain (OES-18)	38 (90)	24 (92)	23 (82)	10 (100)	95 (90)
	Trouble swallowing saliva	42 (100)	26 (100)	28 (100)	9 (90)	105 (99)
	Choked when swallowing	41 (98)	25 (96)	27 (96)	10 (100)	103 (97)
	Dry mouth	38 (90)	20 (77)	25 (89)	9 (90)	92 (87)
	Trouble with taste	39 (93)	23 (88)	22 (79)	8 (80)	92 (87)
	Trouble with coughing	36 (86)	22 (85)	21 (75)	7 (70)	86 (81)
	Trouble with talking	37 (88)	23 (88)	25 (89)	9 (90)	94 (89)

### Relationship between instance of a grade 3–4 toxicity and key PROs at baseline and 2 years

There was no evidence of association between baseline or two-year self-reported physical functioning, role functioning, overall QOL/health or other PRO scales and the instance of a grade 3–4 toxicity, as shown in Additional file 3.

## Discussion

This study reports PROs over two-years for the AGITG DOCTOR trial. PROs were clinically similar across groups by 2 years. Additionally, by 2 years, similar proportions of patients had recovered to within 10 points of baseline for each PRO scale, with the exception of insomnia and pain for the DCR + RT group. The results highlight that among participants who did not respond to early CF, the addition of RT to DCF was associated with some additional burden in the short term. The majority of PRO domains resolved within 24 months of surgery to within 10 points of baseline levels apart from insomnia and pain, and was comparable to that of participants who did respond to early CF prior to surgery.

The pre-surgery time point captures the unique effects of neoadjuvant therapy options for resectable oesophageal adenocarcinoma and offers important data for clinicians to communicate the added burden of these treatment options to future patients faced with a treatment decision, should they not respond to early CF. Non-responders who received an escalation of chemotherapy treatment with DCF and DCF + RT had worse PRO scores for most domains at the pre-surgical time point, which is understandable, given the longer duration of treatment prior to surgery and associated burden and toxicity.

Although no statistical, between-group comparisons were made in this exploratory study due to the sample size (phase 2), the plots suggest that DCF + RT was associated with slightly poorer functional outcomes and side-effects in the short-term. Comparisons between DCF and DCF + RT show that the addition of RT was associated with additional short-term burden, particularly for the fatigue domain, but mean scores were similar across all domains thereafter. The additional short-term burden for the DCF + RT group corresponds to anecdotal reports that participants randomised to the DCF group were able to return to work earlier than participants randomised to DCF + RT. The NeoRes trial (a phase II randomized study comparing neoadjuvant CF chemotherapy with CF CRT) [11] found that the addition of RT to neoadjuvant chemotherapy was associated with worse long-term symptom scores, particularly cough. In contrast, long term PROs from the CROSS trial demonstrated that although health-related quality of life (HRQOL) declined during nCRT, there were no apparent effects of nCRT on postoperative PROs compared with surgery alone [19]. These differences may be due to the less intense chemotherapy and lower RT dosing regimens used in the CROSS trial compared with NeoRes and DOCTOR. This trend warrants further investigation in future studies with larger sample sizes. Importantly, toxicities resolved or returned to within 10 points of baseline levels for most participants and most scales by the end of study follow-up in the DOCTOR trial. DCF + RT was associated with higher proportion of participants achieving major histological response and 5-year overall survival as compared with DCF alone [12]. Future research into patient preferences would be beneficial to determine if the short term negative impact on PROs is acceptable to patients.

The results also highlight the utility of PET scans in discriminating those who respond or do not respond to CF, as baseline PRO scores appear similar across all treatment groups. However CF responders appeared to have better PROs after treatment due to less toxicity. Prior research has suggested that baseline levels of fatigue, reflux [20] and physical symptoms [21, 22] were related to survival outcomes for advanced oesophageal cancers. We explored whether baseline PROs were related to the instance of any grade 3+ adverse events, or to whether the 6-week assessment was completed (assuming non-completion was related to poorer health status), but did not find a relationship. This is similar to Blazeby et al.’s findings that baseline PROs were not predictive of morbidities relating to oesophagectomy or total gastrectomy for cancer of the oesophagus or gastric cardia [17]. Our results also suggest that PROs at 2 years were not impacted by the instance of grade 3+ adverse events, highlighting the importance of collecting PRO data as they capture unique information.

Our study offers important and novel PRO data for neoadjuvant treatments for oesophageal adenocarcinomas; an area where very limited PRO data previously existed. In a randomised study comparing standard esophagectomy to definitive CRT, surgery was associated with an acute decline in several quality of life domains, including reduced physical functioning and global quality of life, and increased fatigue, dyspnoea, coughing; all of which generally improved by 2 years. The effects of CRT were slower to manifest and generally worsened over time [23]. These results may explain the cumulative burden seen for participants randomised to DCT + RT in our study and to nCRT in the NeoRes study [11], although further research is needed to better understand the impact of administering neoadjuvant treatment on patient-centred outcomes. This will be particularly important given the introduction of new therapies such as immune checkpoint inhibitors.

A meta-analysis studying curative treatments for esophageal and gastric cancer, identified 11 studies (few randomized) comparing PROs from patients receiving nCRT or chemotherapy and surgery (*n* = 1015) with surgery alone (*n* = 1021) over the first 12 months postoperatively. Although a clinically-relevant impact of neoadjuvant therapy on PROs was observed prior to surgery, a further decline was seen after surgery in both groups. The HRQOL subscales showed similar scores for surgery alone and neoadjuvant therapy with surgery [24]. In line with these data, the two-year PROs were similar between treatment groups in DOCTOR suggesting most current neoadjuvant regimens are tolerable in the long-term.

This sample was recruited across seven sites, over six-and-a-half years - reflecting the small population with this disease. The Phase 2 design inhibited any reliable statistical comparisons between groups for this number of PRO domains, and larger randomized studies are warranted. Despite the small sample which was appropriate for the primary outcome of histopathological response, the PRO completion rates were excellent overall, but poorest at the 6 week follow up, particularly for DCF and DCF + RT groups. It is fairly typical to see a poor PRO completion rates when PROs are expected to be worse [25], therefore any estimates of PROs at this time point would likely be an underestimate of true (yet unverifiable) PRO values in the populatio﻿n [26, 27]. The results are nonetheless hypothesis generating and provide preliminary data illuminating the lived experience and long term outcomes of neoadjuvant therapy. The instance of some large standard deviation in baseline PRO domain scores is reflective of variability of symptom status in the real world population. A major strength of the PRO analyses in this study is the randomized multicentre treatment allocation and similar baseline scores between treatment arms. Another strength is the use of validated EORTC PRO instruments, which have also been tested for reliability and responsiveness between treatment modalities, and allows comparisons with other PRO studies [11, 19]. An additional limitation is the fact that the most frequently used neoadjuvant regimens are FLOT peri-operative chemotherapy [2] or CROSS-type nCRT, with less intense chemotherapy.

## Conclusions

In conclusion, DOCTOR demonstrated that early metabolic responders to chemotherapy have superior survival outcomes [12] and lower treatment toxicity resulting in less impact on PROs, for patients with resectable oesophageal adenocarcinoma. The administration of DCF + RT to participants who did not respond to early CF was associated with short term detriments to quality of life. However, long-term PROs were similar across groups. A larger proportion of participants did not recover to within 10 points of baseline pain and insomnia scores by 2 years in the DCF + RT compared to other groups. Given that DCF + RT was associated with higher efficacy for non-responders, future patients and clinicians should discuss whether the short-term burden and longer duration of this regimen is personally worthwhile if they do not respond early to chemotherapy. Future studies in oesophageal cancer need to incorporate PROs in order to determine the optimum neoadjuvant strategy.

## Supplementary Information

**Additional file 1.**

**Additional file 2.**

**Additional file 3.**

## Data Availability

Please contact the corresponding author for supporting information or queries.

## Abbreviations

AGITG: The Australasian Gastro-Intestinal Trials Group
DOCTOR: A randomised phase II study of pre-operative cisplatin, fluorouracil and DOCetaxel +/−radioTherapy based on poOR early response to cisplatin and fluorouracil for resectable esophageal adenocarcinoma
CF: Cisplatin, 5 fluorouracil
DCF: Cisplatin, 5 fluorouracil, docetaxel
RT: Radiotherapy
PET: Positron emission tomography
PRO: Patient-reported outcome
