# Supplementary material for: Patient-reported outcome (PRO) results from the AGITG DOCTOR trial: a randomised phase 2 trial of tailored neoadjuvant therapy for resectable oesophageal adenocarcinoma
Source: BMC Cancer. 2022 Mar 15;22:276. doi: 10.1186/s12885-022-09270-4 (PMC8922838; doi:10.1186/s12885-022-09270-4)

**Appendix Table 1.** QOL Completion by group and overall

| Study time point | PET responders: CF | PET non-responders randomised: DCF | PET non-responders randomised: DCF & RT | Others | All patients |
| --- | --- | --- | --- | --- | --- |
| Baseline | 44/44 (100%) | 27/27 (100%) | 32/32 (100%) | 13/13 (100%) | 116/116 (100%) |
| Pre Surgery | 41/44 (93%) | 23/25 (92%) | 25/30 (83%) | 7/10 (70%) | 96/109 (88%) |
| Follow up 6 weeks | 37/42 (88%) | 18/27 (67%) | 20/31 (65%) | 9/11 (82%) | 84/111 (76%) |
| Follow up 12 weeks | 40/42 (95%) | 23/24 (96%) | 27/30 (90%) | 9/11 (82%) | 99/107 (93%) |
| Follow up 6 months | 30/36 (83%) | 18/22 (82%) | 23/27 (85%) | 6/9 (67%) | 77/94 (82%) |
| Follow up 9 months | 28/30 (93%) | 17/20 (85%) | 17/23 (74%) | 4/8 (50%) | 66/81 (81%) |
| Follow up 12 months | 23/30 (77%) | 19/20 (95%) | 18/20 (90%) | 6/8 (75%) | 66/78 (85%) |
| Follow up 24 months | 22/25 (88%) | 11/13 (85%) | 12/13 (92%) | 4/5 (80%) | 49/56 (88%) |

# Post-hoc analysis: Association between baseline PRO scores and PRO assessment completion at week 6.

There is no evidence for any association between PRO scores at baseline and whether or not the participant completed the scheduled PRO assessment at week 6, at a Bonferroni adjusted alpha of α=0.001 .

**Baseline PRO scores by PRO completion at week 6**

|  | | Mean (SD) score at baseline | | Logistic rgeresssion for baseline score as predictor of PRO completion at week 6 | |
| --- | --- | --- | --- | --- | --- |
| QoL instrument | Scale | No week 6 PRO data | Week 6 PRO completed | OR (95% CI) per 10 unit increase in score | P-value |
| QLQ-C30 | Physical functioning | 94 (11) | 94 (10) | 1.09 (0.71, 1.67) | 0.70 |
|  | Role functioning | 83 (20) | 88 (23) | 1.10 (0.92, 1.33) | 0.28 |
|  | Emotional functioning | 72 (24) | 77 (20) | 1.10 (0.90, 1.35) | 0.36 |
|  | Cognitive functioning | 90 (13) | 89 (18) | 0.93 (0.70, 1.24) | 0.62 |
|  | Social functioning | 73 (28) | 81 (26) | 1.11 (0.94, 1.30) | 0.21 |
|  | Fatigue | 25 (18) | 19 (20) | 0.85 (0.68, 1.06) | 0.15 |
|  | Nausea and vomiting | 9 (14) | 12 (19) | 1.11 (0.84, 1.47) | 0.45 |
|  | Pain | 16 (19) | 17 (21) | 1.02 (0.82, 1.27) | 0.85 |
|  | Dyspnoea | 10 (21) | 8 (17) | 0.95 (0.75, 1.20) | 0.65 |
|  | Insomnia | 32 (31) | 25 (29) | 0.92 (0.80, 1.07) | 0.28 |
|  | Appetite loss | 26 (24) | 20 (26) | 0.93 (0.78, 1.10) | 0.37 |
|  | Constipation | 14 (19) | 13 (21) | 0.97 (0.78, 1.20) | 0.78 |
|  | Diarrhoea | 4 (11) | 5 (13) | 1.10 (0.75, 1.61) | 0.63 |
|  | Financial difficulties | 27 (30) | 24 (28) | 0.97 (0.83, 1.14) | 0.73 |
|  | Global health status | 69 (21) | 73 (17) | 1.16 (0.91, 1.46) | 0.23 |
|  | Global Quality of life | 68 (20) | 78 (16) | 1.38 (1.07, 1.79) | 0.01 |
|  | Global health status/QoL | 68 (20) | 76 (15) | 1.29 (1.00, 1.68) | 0.05 |
| OES-18 | Dysphagia | 29 (24) | 22 (20) | 0.87 (0.71, 1.06) | 0.17 |
|  | Problems with eating | 42 (29) | 32 (25) | 0.87 (0.74, 1.03) | 0.11 |
|  | Reflux | 16 (14) | 22 (25) | 1.13 (0.91, 1.41) | 0.27 |
|  | Pain (OES-18) | 23 (20) | 23 (23) | 1.00 (0.82, 1.22) | 0.98 |
|  | Trouble swallowing saliva | 12 (19) | 14 (27) | 1.05 (0.87, 1.27) | 0.60 |
|  | Choked when swallowing | 15 (25) | 14 (23) | 0.97 (0.81, 1.16) | 0.74 |
|  | Dry mouth | 21 (28) | 20 (27) | 0.99 (0.84, 1.16) | 0.89 |
|  | Trouble with taste | 14 (23) | 8 (20) | 0.88 (0.72, 1.06) | 0.18 |
|  | Trouble with coughing | 12 (23) | 11 (22) | 0.99 (0.82, 1.21) | 0.95 |
|  | Trouble with talking | 5 (15) | 4 (14) | 0.95 (0.71, 1.27) | 0.73 |


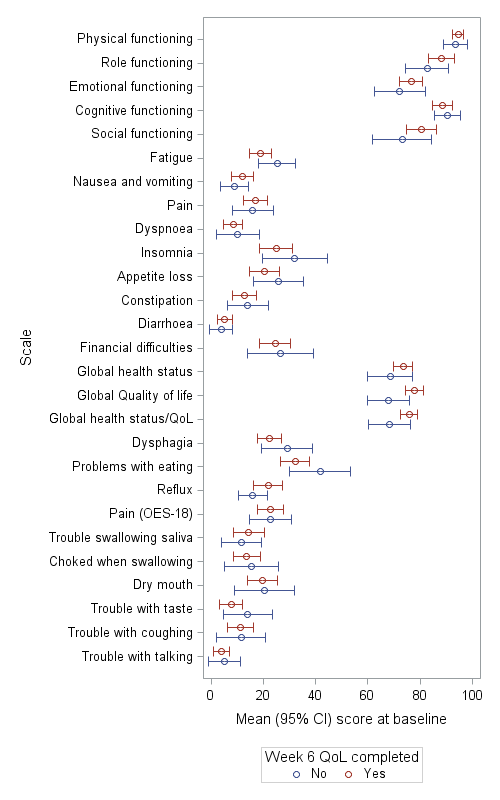

Supplement: Supplementary file 1 — Additional file 1. [file 12885_2022_9270_MOESM1_ESM.docx]
