# Supplementary material for: Patient-reported outcome (PRO) results from the AGITG DOCTOR trial: a randomised phase 2 trial of tailored neoadjuvant therapy for resectable oesophageal adenocarcinoma
Source: BMC Cancer. 2022 Mar 15;22:276. doi: 10.1186/s12885-022-09270-4 (PMC8922838; doi:10.1186/s12885-022-09270-4)
Supplement: Supplementary file 2 — Additional file 2. [file 12885_2022_9270_MOESM2_ESM.rtf]

Appendix 1 – mean PROs over 24 months
(remaining scales not presented in Figure 2)
