# Supplementary material for: Patient-reported outcome (PRO) results from the AGITG DOCTOR trial: a randomised phase 2 trial of tailored neoadjuvant therapy for resectable oesophageal adenocarcinoma
Source: BMC Cancer. 2022 Mar 15;22:276. doi: 10.1186/s12885-022-09270-4 (PMC8922838; doi:10.1186/s12885-022-09270-4)
Supplement: Supplementary file 3 — Additional file 3. [file 12885_2022_9270_MOESM3_ESM.docx]

Appendix 3. Baseline and long-term PROs in participants with and without grade 3-4 adverse events.


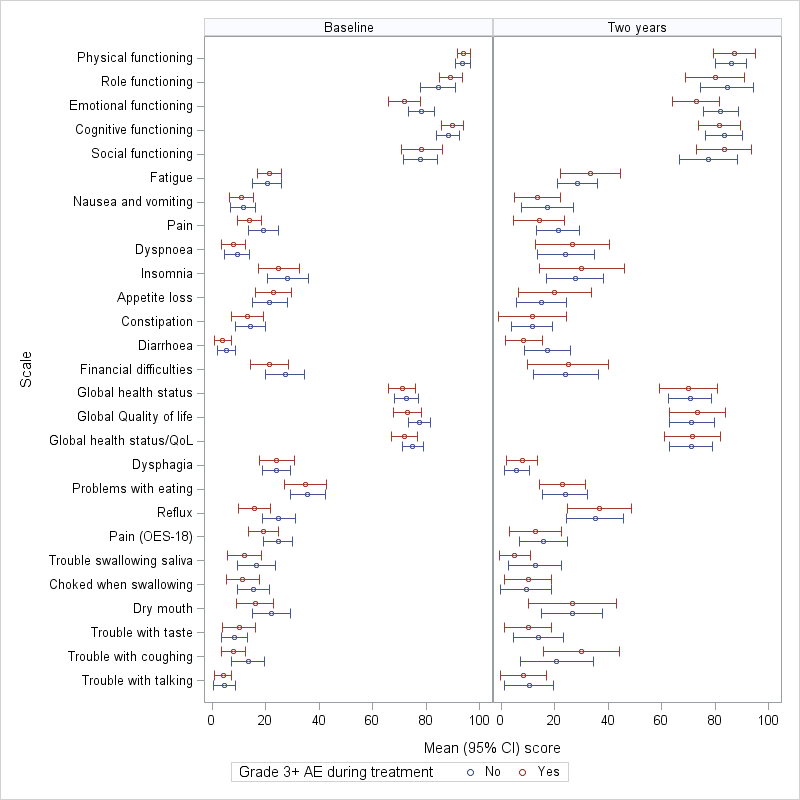


**Association between baseline QoL and Grade 3+ AE during the study**

**Baseline QoL by Grade 3+ AE during study**

|  | | Mean (SD) score at baseline | | Logistic rgeresssion for baseline score as predictor of Grade 3+ AE during study | |
| --- | --- | --- | --- | --- | --- |
| QoL instrument | Scale | No grade 3+ AE during study | Grade 3+ AE during study | OR (95% CI) per 10 unit increase in score | P-value |
| QLQ-C30 | Physical functioning | 94 (12) | 94 (9) | 1.04 (0.73, 1.48) | 0.85 |
|  | Role functioning | 85 (26) | 89 (15) | 1.10 (0.93, 1.32) | 0.27 |
|  | Emotional functioning | 78 (20) | 72 (21) | 0.86 (0.71, 1.03) | 0.11 |
|  | Cognitive functioning | 88 (18) | 90 (15) | 1.07 (0.85, 1.34) | 0.59 |
|  | Social functioning | 78 (26) | 78 (27) | 1.01 (0.88, 1.16) | 0.92 |
|  | Fatigue | 21 (22) | 21 (16) | 1.02 (0.85, 1.23) | 0.82 |
|  | Nausea and vomiting | 12 (19) | 11 (16) | 0.98 (0.79, 1.20) | 0.82 |
|  | Pain | 19 (22) | 14 (16) | 0.86 (0.71, 1.05) | 0.15 |
|  | Dyspnoea | 9 (19) | 8 (16) | 0.96 (0.77, 1.18) | 0.67 |
|  | Insomnia | 28 (31) | 25 (27) | 0.96 (0.85, 1.09) | 0.54 |
|  | Appetite loss | 22 (27) | 23 (24) | 1.02 (0.88, 1.18) | 0.78 |
|  | Constipation | 14 (23) | 13 (21) | 0.97 (0.82, 1.15) | 0.75 |
|  | Diarrhoea | 5 (13) | 4 (11) | 0.92 (0.68, 1.25) | 0.60 |
|  | Financial difficulties | 27 (30) | 21 (25) | 0.93 (0.81, 1.06) | 0.27 |
|  | Global health status | 73 (18) | 71 (18) | 0.95 (0.78, 1.17) | 0.64 |
|  | Global Quality of life | 77 (17) | 73 (18) | 0.86 (0.69, 1.07) | 0.17 |
|  | Global health status/QoL | 75 (16) | 72 (17) | 0.89 (0.71, 1.12) | 0.33 |
| OES-18 | Dysphagia | 24 (21) | 24 (23) | 1.00 (0.84, 1.19) | 0.99 |
|  | Problems with eating | 36 (27) | 35 (27) | 0.99 (0.86, 1.14) | 0.87 |
|  | Reflux | 25 (25) | 16 (22) | 0.83 (0.70, 1.00) | 0.04 |
|  | Pain (OES-18) | 25 (22) | 19 (20) | 0.88 (0.73, 1.06) | 0.17 |
|  | Trouble swallowing saliva | 16 (29) | 12 (22) | 0.93 (0.81, 1.08) | 0.37 |
|  | Choked when swallowing | 15 (24) | 11 (22) | 0.92 (0.78, 1.09) | 0.36 |
|  | Dry mouth | 22 (29) | 16 (25) | 0.92 (0.80, 1.06) | 0.24 |
|  | Trouble with taste | 8 (20) | 10 (22) | 1.04 (0.87, 1.25) | 0.64 |
|  | Trouble with coughing | 13 (26) | 8 (16) | 0.89 (0.73, 1.07) | 0.20 |
|  | Trouble with talking | 5 (17) | 4 (11) | 0.97 (0.75, 1.26) | 0.82 |

**Association between Grade 3+ AE during the study and QoL at 2 years**

**2-year QoL by Grade 3+ AE during study**

|  | | Mean (SD) score at 2 years | |  |
| --- | --- | --- | --- | --- |
| QoL instrument | Scale | No grade 3+ AE during study | Grade 3+ AE during study | P-value from Wilcoxon test |
| QLQ-C30 | Physical functioning | 86 (15) | 87 (17) | 0.55 |
|  | Role functioning | 84 (26) | 80 (23) | 0.33 |
|  | Emotional functioning | 82 (17) | 73 (19) | 0.08 |
|  | Cognitive functioning | 83 (18) | 82 (17) | 0.65 |
|  | Social functioning | 78 (29) | 83 (22) | 0.49 |
|  | Fatigue | 28 (20) | 33 (24) | 0.63 |
|  | Nausea and vomiting | 17 (25) | 13 (18) | 0.91 |
|  | Pain | 21 (21) | 14 (20) | 0.20 |
|  | Dyspnoea | 24 (28) | 27 (30) | 0.79 |
|  | Insomnia | 28 (28) | 30 (34) | 0.95 |
|  | Appetite loss | 15 (25) | 20 (29) | 0.60 |
|  | Constipation | 11 (20) | 12 (27) | 0.66 |
|  | Diarrhoea | 17 (23) | 8 (15) | 0.18 |
|  | Financial difficulties | 24 (32) | 25 (32) | 0.91 |
|  | Global health status | 71 (21) | 70 (23) | 0.89 |
|  | Global Quality of life | 71 (22) | 73 (23) | 0.62 |
|  | Global health status/QoL | 71 (21) | 72 (22) | 0.71 |
| OES-18 | Dysphagia | 6 (12) | 8 (13) | 0.24 |
|  | Problems with eating | 24 (22) | 23 (18) | 0.95 |
|  | Reflux | 35 (28) | 37 (26) | 0.70 |
|  | Pain (OES-18) | 16 (24) | 13 (21) | 0.64 |
|  | Trouble swallowing saliva | 13 (26) | 5 (12) | 0.38 |
|  | Choked when swallowing | 9 (25) | 10 (19) | 0.43 |
|  | Dry mouth | 26 (30) | 27 (35) | 0.80 |
|  | Trouble with taste | 14 (24) | 10 (19) | 0.64 |
|  | Trouble with coughing | 21 (36) | 30 (30) | 0.12 |
|  | Trouble with talking | 10 (24) | 8 (18) | 0.93 |
